# Shifting Demographics among Research Project Grant Awardees at the National Heart, Lung, and Blood Institute (NHLBI)

**DOI:** 10.1371/journal.pone.0168511

**Published:** 2016-12-15

**Authors:** Marc F. Charette, Young S. Oh, Christine Maric-Bilkan, Lindsey L. Scott, Charles C. Wu, Matthew Eblen, Katrina Pearson, H. Eser Tolunay, Zorina S. Galis

**Affiliations:** 1 Vascular Biology and Hypertension Branch, Division of Cardiovascular Sciences, National Heart, Lung, and Blood Institute, Bethesda, Maryland, United States of America; 2 Statistical Analysis and Reporting Branch, Office of Planning, Analysis and Communication, Office of Extramural Research, National Institutes of Health, Bethesda, Maryland, United States of America; 3 Office of Public Health Scientific Services, Centers for Disease Control and Prevention, Atlanta, Georgia, United States of America; Harvard Medical School, UNITED STATES

## Abstract

The present study was initiated because of concerns expressed by NHLBI-funded mid-career investigators regarding perceived difficulties in the renewal of their grant awards. This led us to ask: “Are mid-career investigators experiencing disproportionate difficulties in the advancement of their professional careers?” Our portfolio analysis indicates that there has been a significant and evolving shift in the demographics of research project grant (RPG) awardees at NHLBI. In 1998, mid-career (ages 41–55) investigators constituted approximately 60% of all investigators with the remaining 40% being equally divided between early-stage (ages 24–40) investigators and established (ages 56 to 70 and older) investigators. However, since 1998, the proportion of established RPG awardees has been increasing in a slowly progressive and strikingly linear fashion. At the same time the proportion of early-stage awardees fell precipitously until 2006 and then stabilized. During the same period, the proportion of mid-career awardees, which had been relatively stable through 2006, began to fall significantly. In examining potential causes of these demographic shifts we have identified certain inherent properties within the RPG award system that appear to promote an increasingly more established awardee population and a persistent decrease in the proportion of mid-career investigators. A collateral result of these demographic shifts, when combined with level or declining funding, is a significant reduction in the number of RPG awards received by NHLBI mid-career investigators and a corresponding decrease in the number of independent research laboratories.

## Introduction

The National Heart, Lung, and Blood Institute (NHLBI) is one of 27 independent institutes and centers at the National Institutes of Health (NIH). It is the third largest institute, by appropriation level, with an annual yearly budget of more than $3 billion dollars. NHLBI’s mission is to provide global leadership for research, training, and education programs to promote the prevention and treatment of heart, lung, blood, and sleep disorders and diseases and to enhance the health of all individuals so that they can live longer and more fulfilling lives.

**Fig 1:**
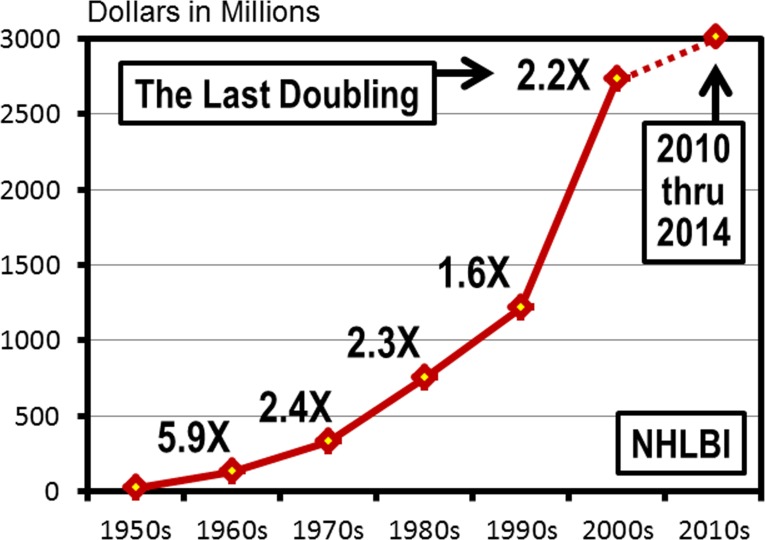
Five Decades of Exponential Growth in Congressional Appropriations at NHLBI. Average yearly congressional appropriation per decade for the National Heart, Lung, and Blood Institute from 1950 through 2014 [1]. The numbers adjacent to the data points indicate the magnitude of appropriation increase relative to the previous decade.

The NHLBI grew and evolved in the midst of an extraordinary funding period. The first five decades (1950–2000) were characterized by exponential increases in congressional funding appropriations [1] (Fig 1). However since the early 2000s the funding levels for the NHLBI and NIH, adjusted for inflation, have been declining [2]. This funding decline has placed significant constraints on the number of grants that can be awarded to the biomedical research community, the result being that while the number of applicants has been steadily increasing during this period the number of awarded grants has remained static [3]

The primary vehicle by which NHLBI (and the NIH generally) funds biomedical researchers is the Research Project Grant (RPG) award system, under which monies are distributed to scientists to study and treat heart, lung, blood, and sleep disorders and diseases. The RPG award system relies, for the most part, on proposals from individual investigators to study various aspects of normal biology and the diseases and disorders of abnormal biology. A peer system reviews these proposals and ranks them according to scientific merit and likely biomedical impact. Many, though not all, of the RPG funding mechanisms are ranked by percentile, a system which allows many different proposals evaluated by many different qualified individuals, to be pooled and approximately compared.

**Fig 2:**
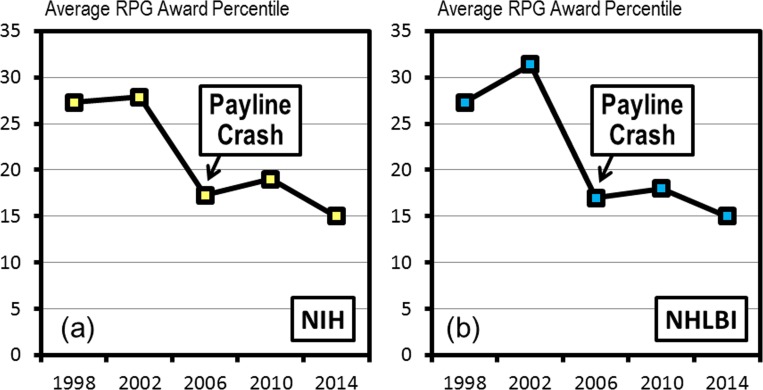
The Percentile Payline Crash of 2006. Average aggregate payline percentiles, for those RPG grant mechanisms that were percentiled, for select years between 1998 and 2014 at (a) NIH and (b) NHLBI.

**Fig 3:**
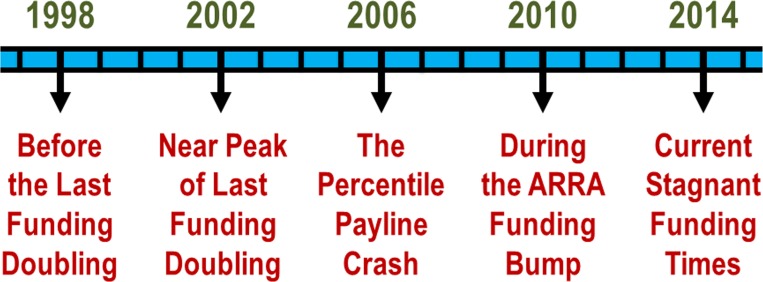
Analysis Timeline. Timeline of funding and payline events during analysis project period.

Shortly after the last funding doubling (that peaked in 2003 [2]) the percentile scores payline, which determines which projects will receive biomedical research funding, crashed at the NHLBI and at the NIH and bottomed out in 2006 (Fig 2). The reverberations of that crash are still being felt among the biomedical research community today and may be a contributing factor to the difficulties now being faced by mid-career investigators. This present study examines the demographics of the NHLBI research community prior to the percentile payline crash of 2006 and the period immediately afterward. In particular, we are attempting to understand the underlying causes of certain shifts in the demographics of RPG awardees during the period of 1998 through 2014 (see analysis timeline in Fig 3).

## Methods and Analysis

The primary data, from which most of the figures, graphs, and tables were derived, are from the Statistical Analysis and Reporting Branch of the Office of Planning, Analysis and Communication within the Office of Extramural Research at the National Institutes of Health. The data consist of summaries of NHLBI and NIH RPG direct costs, number of awards, number of applications, number of awardees, and direct costs per awardee. The data are further broken out by year, age group, and total awards vs. competing awards. Competing awards are either new awards or renewals of existing awards that require competitive peer review. Total awards are a combination of competing awards and non-competing awards (which are awards within a previously approved project period for which a recipient does not have to compete with other applicants). We also have utilized RPG award rates, which is the number of competing awards divided by the number of competing applications.

The RPG award system has many different funding mechanisms to support different types of project proposals. During the time frame of this study there were 43 different funding mechanisms utilized in three broad categories (R, P, and U mechanisms [4]). Unless otherwise specified, our analysis reflects RPG awards as an aggregate whole with the exception of the exclusion of small business focused grants (SBIR and STTR awards). Data about grant applications and awards is maintained in the Information for Management, Planning, and Analysis and Coordination II (IMPACII) database. The data for the present study represent extramural activities for applications submitted by grantee organizations for fiscal years 1998 through 2014. In 2006, the NIH began to recognize multiple principal investigators (MPIs) [5]. For those applications, there is a single contact principal investigator (designated PI) and then additional PIs (designated MPIs). All PIs, including the MPIs, are included in this analysis. In those instances when we are examining “direct dollars per PI per year”, the total direct dollars of a multi-PI award is divided by the total number of PIs (including those designated as MPIs).

In this study we used the age of the RPG awardees as a proxy for their career stage. We divided the RPG awardee population into three groups each spanning approximately 15 year intervals: the first group taken to represent early-stage investigators included ages 24–40, the mid-career investigators group comprised ages 41–55, and the established investigators group covered ages 56–70 and above. This arbitrary categorization allowed us to eliminate the variability associated with other categorization schemes, such as academic title or time from terminal degree, and established a rigid standard for comparison purposes. It should be noted that birthdate is a voluntary item collected as a part of a person’s role record or profile and it was used to calculate age at the time of application or award. Investigators with withheld or unknown age are not included this analysis.

During the time course of this study RPG awardees are, of course, migrating from one age group into another. We should point out that we did not have data on specific individuals and therefore we are not able to track specific cohorts. This study is mostly about taking a snapshot every four years and looking to see what the current distribution of the awardee population looks like at those time points and then trying to understand some of the underlying causes driving the demographic shifts. We have chosen to use proportion as the primary, though not exclusive, measure of the population groups because it allows for direct comparison of population group distribution at the different time points eliminating the great variability associated with significant funding level changes (before the doubling, after the doubling, during the American Recovery and Reinvestment Act (ARRA) funding “bump”) and with significant changes in percentile paylines.

To calculate the average number of research project grant awards per awardee (1998–2014), the total number of awards for each awardee during the fiscal year(s) while they belonged to a given age group of the award receipt was summed, and then a Tukey-Kramer least squares means test was computed for a given year, and for all years (1998–2014). We also performed a second type of statistical analysis on this data. That analysis looked at the odds ratios of having more than one grant among the age groupings. We compared groups of investigators with one award against those investigators with more than one award, in each age group. The results of the odds ratio analysis and the Tukey-Kramer least squares analysis were similar and only one (the Tukey-Kramer analysis) is shown. All statistical analyses were performed using SAS version 9.3 for Windows (SAS Institute Inc., Cary, NC).

## Results

### Shifting Demographics among RPG Awardees

**Fig 4:**
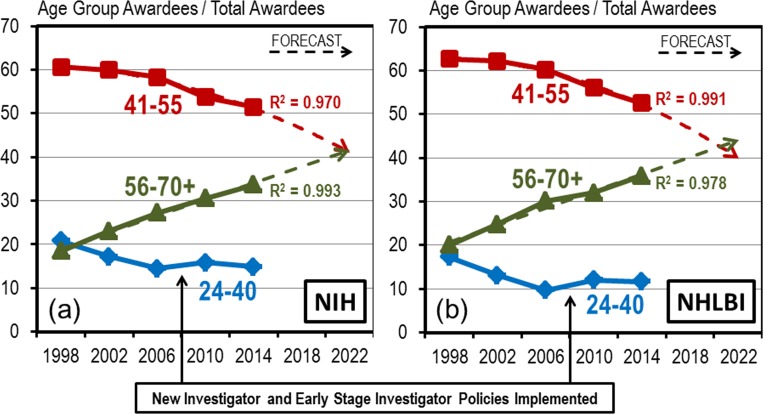
Demographics of RPG Awardees is Shifting towards an Older Population. Proportion of RPG awardees by age group (blue diamond = ages 24–40, red square = ages 41–55, green triangle = ages56-70+) for select years between 1998 and 2014 for (a) NIH and (b) NHLBI. Dashed lines are trend line forecasts through the data curves with R^2^ values appended.

In 1998, mid-career (ages 41–55) investigators constituted approximately 60% of all NHLBI investigators with the remaining 40% being equally divided between early-stage (ages 24–40) investigators and established (ages 56 to 70 and older) investigators (Fig 4B). However, since 1998, the proportion of established RPG awardees has been increasing in a slowly progressive and strikingly linear fashion. At the same time the proportion of early-stage NHLBI RPG awardees fell precipitously until 2006 and then stabilized post 2006. During the same period, the proportion of mid-career NHLBI RPG investigators, which had appeared to be relatively stable through 2006, began to fall significantly.

These shifts in demographics are not specific to NHLBI but are indicative of broader patterns at the NIH generally (Fig 4A). Trend line forecasts projected through the data points suggests that there could be a significant reversal in the relative proportions of mid-career and established investigators by 2020 at the NHLBI and 2022 at the NIH. The overall aging of the biomedical research workforce has been well documented by others [6, 7], as have been the career difficulties faced by younger investigators [8–10]. However, the underlying causes that are driving the shifting demographics remain somewhat uncertain. We have considered several possible hypotheses including potential inability of younger scientists to compete with more experienced investigators and potential bias in the review process that might favor more established scientists. We also have observed that the patterning of the demographic changes suggests the possibility that inherent properties within the RPG system itself could be conferring advantage or disadvantage to certain subpopulations of the biomedical research community.

### Characteristics Associated with Underlying Causes

There are certain characteristics associated with potential underlying causes of the demographics shifts that can be inferred from the shapes and timing of the data curves. First, the overall demographic shift is small, approximately 1% per year (note the rate of the linear rise of the established investigator curves in Fig 4). Second, the underlying affect is remarkably constant over time in spite of significant variability in the level of funding appropriations and paylines. The rise in the proportion of established investigators began before the most recent doubling of the NIH/NHLBI budgets (1998), continued unperturbed through the budget doubling (2002), proceeded in a linear fashion through the percentile payline crash (2006), was unaffected by the ARRA funding bump (2010) [2], and finally was still rising linearly through during the current stagnant funding period (2014) [2].

The third characteristic of the potential cause(s) of this demographic shift is directionality, in that the cause(s) demonstrably push the demographic towards a more established investigator awardee population. And fourth, the causes are imbedded in the RPG award system. This latter point is more assumption than inference. However, we will present data that suggests inherent aspects of the NIH RPG system contain properties that could confer enhanced survival benefits towards a more established investigator demographic. These properties, which were once considered to be unimportant or at least less important when the budget was continually rising, now during this period of level or declining funding, are causing significant challenges for NHLBI awardees.

### Success and Failure within the RPG System

**Fig 5:**
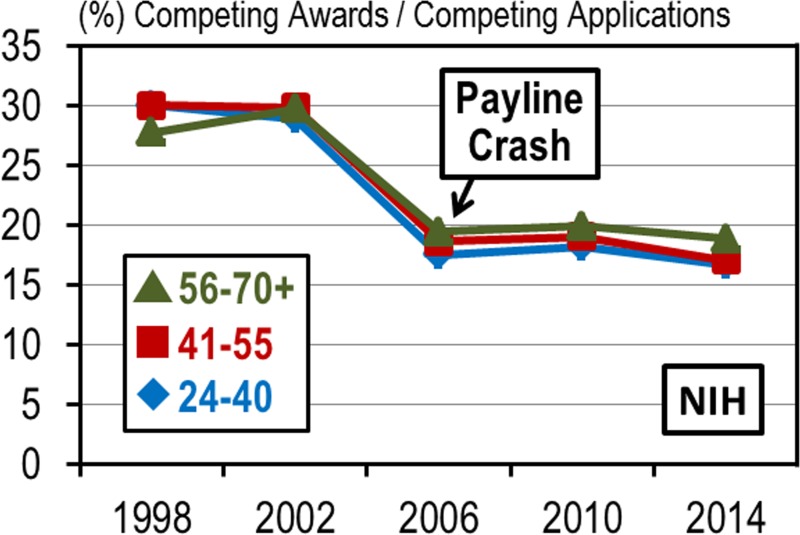
Prospects of Receiving a New Award or Renewing an Existing Award are Approximately Equal among the Age Groups. RPG competing award rates at NIH by age group for select years between 1998 and 2014.

At its most basic level, the RPG system functions to award new grants or, when significant progress is demonstrated, renew existing grants. Review of the competing RPG award rate data from 1998 through 2014 indicates that most applicants, regardless of age, have an approximately equal chance of winning a new award or renewing an existing award (Fig 5). This is an important finding because NIH is dedicated to distribution of research funding dollars by mechanisms that are fair and balanced and firmly based on the merits of the proposals. The finding also implies that shifting demographics are not due to the inability of younger scientists to adequately compete with more established investigators because all of the age groups performed approximately the same. Even when the percentile paylines crashed, the award rates for all age groups remained similar. Fig 5 also provides evidence that the shifting demographics are not caused by systematic bias in the review process that favors more established investigators because if there were systemic bias then the award rates would reflect that.

The absence of visible reviewer bias in favor of more established investigators and the recognition that the different age groups appear to be competing in a similar manner has led us to explore alternative causality possibilities. In particular we are considering the differential effects of success and failure within the RPG award system because, while the prospects for success may be approximately equal, the consequences of failure, particularly the failure to renew a grant award, are not equal. Consider this scenario: when two academic researchers, one with only one grant and the other with multiple grants, compete for a grant renewal and neither receives the award renewal, then the investigator with only one grant may be forced out of the academic research community because salaries (“soft” money) are linked to grant awards. The investigator with multiple grants will be able to re-adjust the allocation of soft money salary to the remaining grant(s) and will, thereby, remain in the system.

**Fig 6:**
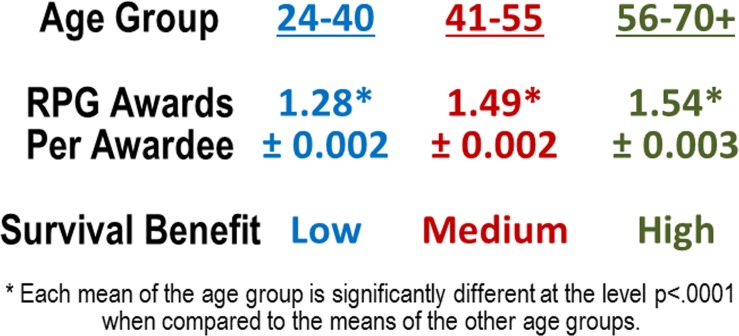
Gradient of Multi-Grant Associated Survival Benefit. The aggregate average number of the total NIH RPG awards per awardee by age group plus or minus the standard error of the mean during the period of 1998 through 2014.

Multiple grant awards, therefore, confer a form of “enhanced survival benefit” over those investigators who have only a single grant. This unintended survival benefit within the RPG award system can be estimated by calculating the average number of RPG awards per awardee in each of the age groups (Fig 6). There is a gradient of multi-grant associated survival benefit that extends from the youngest investigators with the lowest survival benefit to the more established investigators with the highest.

**Fig 7:**
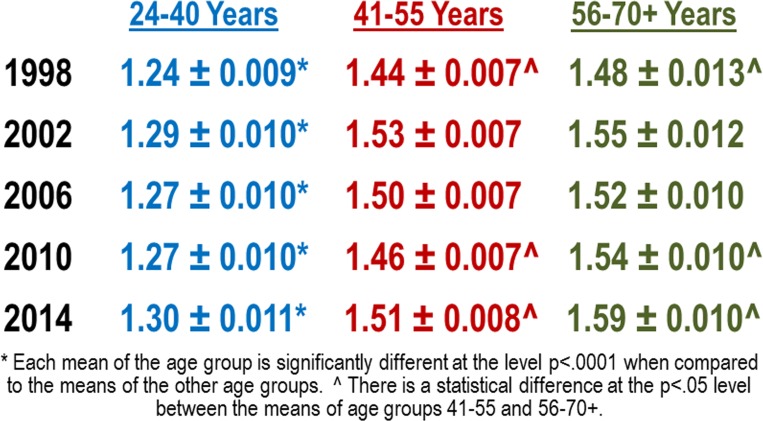
Magnitude of Multi-Grant Survival Benefit is Approximately Constant over Time. Average number of NIH RPG awards per awardee by age group plus or minus the standard error of the mean for select years between 1998 through 2014.

The gradient of multi-grant associated survival benefit has been remarkably stable over time (Fig 7). The magnitude of the survival benefit, as estimated by the average number of grants per investigator, was approximately the same in 1998 as it was in 2014. The magnitudes were unaffected by the doubling of the congressional NIH/NHLBI appropriations in the early 2000s and were unaffected by the payline crash of 2006. These small but stable differences in enhanced survival benefits could be one of the underlying causes of the shifting demographics.

There is yet another component that also contributes to enhanced survival benefit and that is “hard” salary money. Investigators whose salaries are paid by university/medical center funds (such as endowed chairs or professorships) are afforded an additional measure of survival benefit. The age distribution of those academic research investigators who have endowed chairs or professorships is not known, but we assume that investigators with hard salary monies tend to be more established investigators. Thus, the aggregate enhanced survival benefit consists of the multi-grant survival benefit and the hard-money survival benefit which, combined, form a gradient of advantage from the youngest investigators with the least advantage to established investigators with the highest advantage.

In the years between 1998 and 2006, the population with the least survival benefit was the early-stage investigator group (ages 24–40) and indeed they experienced the largest proportional declines (Fig 4). During the same period the population with highest survival benefit, the established investigators (ages 56–70 and older) experienced the largest, and almost reciprocal proportional increases. The population in the middle (the mid-career investigators, ages 41–55) with the mid-level survival benefit was approximately stable.

Post 2006, changes were made to the RPG award system with the introduction of the New Investigator and Early Stage Investigator policies [11] which made it easier for young investigators to secure their first significant grant. This, we believe, resulted in the stabilization of the early investigator population post 2006. The net effect, however, seems to have been a transfer of the demographic losses from the early stage investigators to the mid-career investigators. Indeed, in the period from 2006 to 2014, when the mid-career and established investigator populations were the only population groups competing with the same grant award rules, the mid-career investigators experienced the largest proportional losses and the established investigators had the largest and almost reciprocal proportional gains.

### Disproportionate Allocation of RPG Direct Dollars

**Fig 8:**
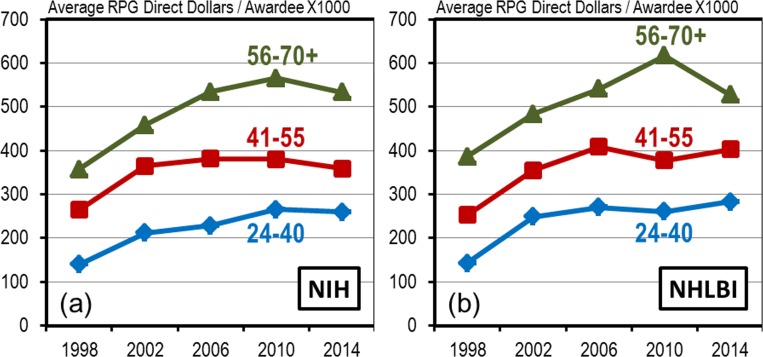
Disproportionate Allocation of Total RPG Direct Dollars. **A**verage amount of total RPG direct dollars per awardee by age group for selected years between 1998 and 2014 at (a) NIH and (b) NHLBI.

In addition to the enhanced survival benefit there is another factor that plays a role in the shifting demographics. That factor is the disproportionate allocation of RPG direct dollars (Fig 8). Ever since 1998 and through 2014 the NIH and NHLBI have distributed significantly more RPG direct dollars to established investigators, on an average direct dollar per investigator basis, than to all other groups. In contrast early stage investigators received the least amount of direct dollar funding and the mid-career investigators received an intermediate level.

**Fig 9:**
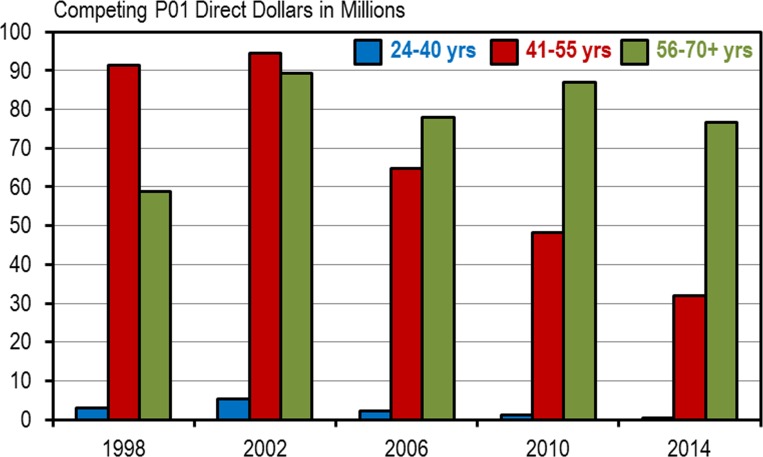
Changing Distribution of NIH P01 Funding. Competing P01 direct dollars by age group (blue bar = ages 24–40, red bar = ages 41–55, green bar = ages 56–70+) for select years between 1998 and 2014 at NIH.

This of course is not surprising. As we have already noted, established investigators generally have more RPG awards and those awards tend to be larger awards. The disproportionate allocation of direct dollars is also enhanced by certain RPG funding mechanisms that have become, over time, heavily weighted towards established investigators. One example is the program project grant P01 award mechanism [4]. At NIH, between 1998 and 2002, most of the P01 direct dollars were awarded to mid-career investigators, but rapidly and steadily over the next 12 years the P01 awards began to be dominated by established investigators (Fig 9). By 2014 established investigators, who represented just 34% of the total NIH RPG awardee population, were receiving 70% of the competing P01 direct dollars. The situation is much the same at NHLBI. In 2014 NHLBI established investigators, who represented 36% of the total RPG awardee population, received 78% of the competing P01 direct dollars.

The disproportionate allocation of direct dollars affects the shifting demographics in the following fashion: as the proportion of established investigators gradually increases the pool of remaining RPG monies becomes disproportionately smaller. The pool becomes disproportionately smaller because established investigators on average receive substantially more direct dollars per investigator than the other age groups. Thus, the capacity of the RPG system to fund less expensive mid-career and early-stage investigators is being disproportionately eroded in favor of more expensive established investigator laboratories. And, because the proportion of early-stage investigators has been stabilized, most of the downward pressure on funding is now being focused on mid-career investigator directed laboratories.

### Consequences of Stagnant Funding Levels

The enhanced survival benefit selectively reduces the survival probabilities of younger investigators and drives the RPG awardee demographic towards a more established investigator population. The disproportionate distribution of direct dollars augments the enhanced survival benefit by selectively accelerating the loss of mid-career investigators and thus also contributes towards a more established investigator demographic. The confluence of the enhanced survival benefit and the disproportionate dollars affect, when combined with overall funding levels that are flat or decreasing, has created a crisis among NHLBI mid-career investigators.

**Fig 10:**
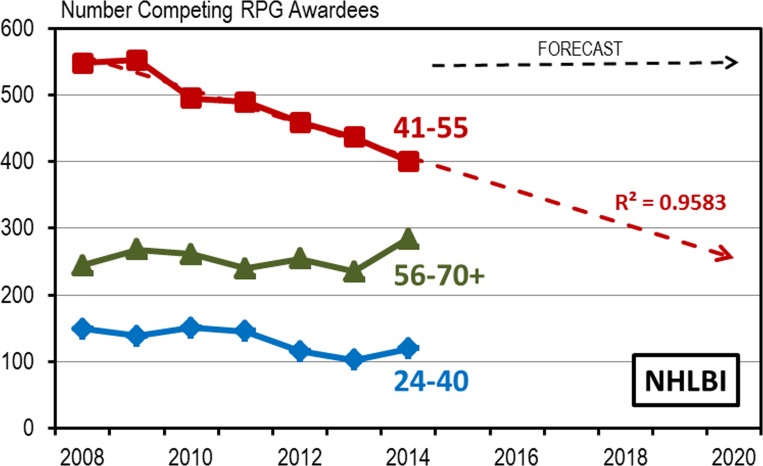
Selective Reduction in the Number of Mid-career Awardees. Number of NHLBI competing RPG awardees by age group for selective years between 1998 and 2014. Dashed line is a trend line forecast through the data curve with R^2^ value appended.

The first five years of the 2010 decade (2010–2014), when mapped onto the graph of NHLBI congressional appropriations, shows the dramatic turn away from exponential growth (Fig 1) and, when adjusted for inflation, the overall funding levels have been declining [2]. In the five year period between 2010 and 2014 the number of competing awards received by NHLBI mid-career investigators declined significantly (Fig 10). By 2014 there were 155 fewer grants awarded to conduct heart, lung, blood, and sleep research than there were in 2009. During this same period the number of established and early-stage competing awardees remained relatively stable. Virtually all of the losses (152 out of 155) have occurred among the ranks of the mid-career investigators. The downward trend of the number NHLBI mid-career investigator awards is linear and projects a substantial additional reduction in the number of mid-career investigator RPG awards by 2020.

## Discussion

We do not know for certain that the enhanced survival benefit or the disproportionate dollars affect are the primary drivers of the demographics shifts or that they, in combination with flat or declining funding, are the causes of the selective decrease in mid-career investigator awards at NHLBI. This scenario has inferential plausibility but, given the nature of the system, it may never be possible to firmly establish definitive cause and effect. What does appear certain however is that at the NHLBI there has been a significant and evolving shift in the demographics among RPG awardees towards an older population and that, more recently, there has been a significant and selective reduction in the number of awards to mid-career investigators and a corresponding reduction in the proportion of mid-career investigator directed laboratories. In the absence of substantive changing circumstances both trends seem likely to continue.

NHLBI has begun to address some of these issues. In 2015, the Institute began to make greater use of Selective Pay [12] which, among other criteria, can provide support to productive laboratories that are running out of funds but have not yet been able to reach the current percentile funding threshold for a new award or renewal of an existing award. The Institute also has begun to utilize the R56 mechanism [13] to provide bridge funding for one year while investigators gather more data to revise and resubmit their competing grant applications. In addition NHLBI has recently established a new funding mechanism, the R35 [14], which is intended to provide more stable long-term funding for investigators who meet several criteria.

There are other remedial approaches that could be taken. Limits on the number of RPG awards per awardee could be instituted and/or a cap could be placed on the total amount of direct dollars allocated per investigator per year. Both of these approaches have practical and philosophical difficulties. How would the “right” number of RPG awards be empirically determined? If an investigator is exceptionally productive why should there be any limitations? Given the wide range in project costs, how would a direct dollar cap be structured? And finally, would the Institute actually be better served with policies that promote a more stable distribution of investigators? NHLBI is currently on a trajectory that is resulting in fewer, more expensive (on average) laboratories that are directed by a population of very experienced investigators. This trend may decrease the diversity of independent investigators and may also decrease the diversity of scientific approaches to many important scientific problems. There is some evidence, from another NIH institute, that suggests “that supporting a greater number of investigators at moderate funding levels is a better investment strategy than concentrating high amounts of funding in a smaller number of researchers” [15]. Of course, the mission of each institute is uniquely its own and the “best” strategy for one may not be the best strategy for all.

The shift in demographics towards a more established investigator population, if caused by the enhanced survival benefit and/or the disproportionate dollars affect, will be difficult to correct within the current RPG structures. The RPG award system is a patchwork of mechanisms and policies that for the most part was cobbled together during the five decade period in which congressional appropriations for NHLBI were increasing exponentially. It was never conceived or designed as an integrated whole and if there are structural elements within the RPG system that confer benefits to one age group over another, they will be challenging to address. A major part of the problem is that attempts to correct disparities with one age group will inevitably disadvantage others. We believe this is what likely happened with the attempt to stabilize the losses in the early investigator population after the payline crash of 2006. The net effect of which seems to have been to transfer the losses to the mid-career investigators. NHBLI faces a difficult task in trying to eliminate structural disparities and establish a stable investigator pipeline.

One objective of this report is to expand the discussion of “the aging of the scientific workforce” from some commonly held beliefs to some, potentially, alternative explanations. For instance, a widely held belief within the academic research community is that the scientific workforce is aging because more established investigators are simply better scientists. In this belief we are all “Darwinists”, in that, during stressful times our first presumption is that the best survive and the merely good fall away. But what if that is not the full situation? What if there are small incremental survival benefits that have given more established investigators an extended timeframe to remain within the academic research community and, thereby, the time needed to secure those increasingly hard to get grant opportunities? Perhaps the survival benefits and disproportional funding didn’t make much of a difference when the congressional appropriations were doubling every ten years and the paylines were higher but now, with stagnant funding and low paylines, they are having a much more magnified effect. We are, in this report, advancing the hypothesis that the RPG system itself contains structural properties that are gradually and steadily creating demographic changes that, in turn, are diminishing the ability of NHLBI and the NIH to maintain a stable pipeline of investigators.
